# Monogenic diabetes in New Zealand - An audit based revision of the monogenic diabetes genetic testing pathway in New Zealand

**DOI:** 10.3389/fendo.2023.1116880

**Published:** 2023-03-24

**Authors:** Francesca Harrington, Mark Greenslade, Kevin Colclough, Ryan Paul, Craig Jefferies, Rinki Murphy

**Affiliations:** ^1^ Diagnostic Genetics, Department of Pathology and Laboratory Medicine, Te Whatu Ora – Health New Zealand, Te Toka Tumai Auckland, Auckland, New Zealand; ^2^ Exeter Genomics Laboratory, Royal Devon University Healthcare National Health Service (NHS) Foundation Trust, Exeter, United Kingdom; ^3^ Te Huataki Waiora School of Health, University of Waikato, Hamilton, New Zealand; ^4^ Starship Children’s Health, Te Whatu Ora – Health New Zealand, Te Toka Tumai Auckland, Auckland, New Zealand; ^5^ The Liggins Institute, The University of Auckland, Auckland, New Zealand; ^6^ Department of Medicine, University of Auckland, Auckland, New Zealand

**Keywords:** monogenic diabetes, New Zealand, MODY, genomics, genetic testing, Aotearoa (New Zealand)

## Abstract

**Aims:**

To evaluate (a) the diagnostic yield of genetic testing for monogenic diabetes when using single gene and gene panel-based testing approaches in the New Zealand (NZ) population, (b) whether the MODY (Maturity Onset Diabetes of the Young) pre-test probability calculator can be used to guide referrals for testing in NZ, (c) the number of referrals for testing for Māori/Pacific ethnicities compared to NZ European, and (d) the volume of proband vs cascade tests being requested.

**Methods:**

A retrospective audit of 495 referrals, from NZ, for testing of monogenic diabetes genes was performed. Referrals sent to LabPlus (Auckland) laboratory for single gene testing or small multi-gene panel testing, or to the Exeter Genomics Laboratory, UK, for a large gene panel, received from January 2014 – December 2021 were included. Detection rates of single gene, small multi-gene and large gene panels (neonatal and non-neonatal), and cascade testing were analysed. Pre-test probability was calculated using the Exeter MODY probability calculator and ethnicity data was also collected.

**Results:**

The diagnostic detection rate varied across genes, from 32% in GCK, to 2% in *HNF4A*, with single gene or small gene panel testing averaging a 12% detection rate. Detection rate by type of panel was 9% for small gene panel, 23% for non-neonatal monogenic diabetes large gene panel and 40% for neonatal monogenic diabetes large gene panel. 45% (67/147) of patients aged 1-35 years at diabetes diagnosis scored <20% on MODY pre-test probability, of whom 3 had class 4/5 variants in *HNF1A*, *HNF4A* or *HNF1B*. Ethnicity data of those selected for genetic testing correlated with population diabetes prevalence for Māori (15% vs 16%), but Pacific People appeared under-represented (8% vs 14%). Only 1 in 6 probands generated a cascade test.

**Conclusions:**

A new monogenic diabetes testing algorithm for NZ is proposed, which directs clinicians to choose a large gene panel in patients without syndromic features who score a pre-test MODY probability of above 20%.

## Introduction

Monogenic forms of diabetes are estimated to account for around 1-4% of childhood diabetes, with distinct therapeutic implications from the more common type 1 and type 2 diabetes mellitus (1). However the diagnosis of monogenic diabetes remains a significant challenge and a study in the UK estimated >80% of monogenic diabetes is undiagnosed, largely due to variation in access for referrals for genetic testing (2).

Historically called Maturity Onset Diabetes of the Young (MODY), the classic triad was diagnosis under 25 years, a family history of diabetes with autosomal dominant inheritance and minimal/no requirement for insulin. However, an increasing list of monogenic diabetes genes and associated clinical features, combined with increased awareness and rising levels of type 2 diabetes, mean this triad is not as relevant today for selecting people who may benefit from genetic testing (3).

In Aotearoa New Zealand, there is publicly funded access to genetic testing for monogenic diabetes through endocrinologists. National guidelines for monogenic diabetes genetic testing from the New Zealand Society for the Study of Diabetes (NZSSD) require clinical identification of potentially affected individuals based on the absence of typical features for type 1 diabetes (i.e. absence of auto-antibodies, persistence of significant C-peptide) or type 2 diabetes (e.g. absence of obesity, hypertension, hyperlipidaemia) (4). In 2013, guidance was published on using a clinical phenotype-driven approach, where clinicians were directed to look for key features to identify single genes to test (5). For example, neonatal onset (directed neonatal diabetes gene panel testing); retrospective mild fasting hyperglycaemia (characteristic of *GCK*); high HDL *(HNF1A*); neonatal macrosomia (*HNF4A)* and syndromic features present in patients with either *HNF1B* or mitochondrial forms (MIDD) (5). In 2018, an updated guideline was released. Under this guideline, testing may be performed using single gene tests, if characteristic features are present for *GCK, HNF1A/HNF4A, HNF1B*, or mt.3243A>G; or alternatively, use a gene panel if characteristics are not clearly a single monogenic phenotype. This may be either as a small multi-gene panel (any combination of the 4 single genes) or a large gene panel, available overseas (6). Two different large gene panels are available – either a general monogenic diabetes panel, or a neonatal diabetes panel. The comparative diagnostic yield for monogenic diabetes testing using the clinical phenotype-guided single gene approach, compared to the small multi-gene panel testing approach or large gene panel testing has not previously been studied in the NZ population.

A clinical prediction model (MODY probability calculator) has been developed previously, using 594 Europeans with *HNF1A, HNF4A* or *GCK* monogenic diabetes and 597 with types 1 and type 2 diabetes, and can be used to derive the pre-test probability of monogenic diabetes (7). This calculator uses simple characteristics such as age at diagnosis, sex, time to insulin treatment, glucose lowering treatment, body mass index (BMI), HbA1c and family history of diabetes. The logistic regression model assumes a background prevalence of monogenic diabetes of 4.6% and can be used for individuals diagnosed with diabetes below 35 years of age and of European ethnicity. A >20% risk score for patients not on insulin, or >10% if insulin treated, is currently used in the United Kingdom, and referrals to the Exeter laboratory that include a calculation of the MODY probability score have a higher positive test rate than those that do not (33% vs 25%) (8). However, there is no data on the prevalence of monogenic diabetes in NZ and it is not known what MODY pre-test threshold would be optimal for use in the NZ population. This is particularly of note given the high prevalence of young-onset type 2 diabetes seen in people of Māori and Pacific ethnicities, making the NZ population clinically different to that of the UK (9).

Furthermore, the current testing algorithm relies on clinician judgement around atypical features for type 2 diabetes, which is more common and earlier in onset among Māori and Pacific peoples relative to people of European ethnicity. Hence, it is unclear whether testing volume and diagnostic rates for monogenic diabetes are different among Māori and Pacific people relative to other ethnicities.

Our first aim was to describe what is currently known about single gene versus small gene panel or large gene panel testing for monogenic diabetes in NZ, through evaluating the detection rate by gene and test type for all referrals for monogenic diabetes testing across NZ, between 2014 and 2021. Our second aim was to test whether the MODY probability calculator (7) could be used to guide patient selection for those without neonatal onset of diabetes or other syndromic forms of diabetes. Our third aim was to investigate whether testing frequency differed across ethnicities, particularly for Māori and Pacific populations. Fourthly, it is known that the highest yield for monogenic diabetes testing is in cascade testing, hence we aimed to examine the volume of cascade testing relative to proband testing. Finally, the information gathered in this audit was used to inform a new testing algorithm for monogenic diabetes in NZ.

## Materials and methods

All monogenic diabetes genetic test requests made by NZ endocrinologists between 01/01/2014 – 31/12/2020, were captured by searching the LabPlus laboratory (Auckland District Health Board, NZ) in house test database and the Exeter Genomics Laboratory (Royal Devon & Exeter Hospital, UK) in house database. Patients must have been suspected as having monogenic diabetes after assessment by a consultant endocrinologist and hence referred for genetic testing from any medical service location across NZ. Genetic testing for monogenic diabetes was publicly funded for all NZ residents.

Data for gene(s) tested, testing method, and result, as well as age, gender, and requestor was identified from test records for all patients. Where available, requestor provided information on BMI, age at diabetes diagnosis, history of diabetic ketoacidosis (DKA), time to insulin treatment, HbA1c and family history of a parent with diabetes was noted. Where sufficient clinical data was available, either on the request card or from the medical notes, and patients were aged 1-35 years at time of diagnosis, the MODY pre-test probability was calculated as per the model used by Shields et al. (7, 10).

LabPlus was the only laboratory in New Zealand offering testing of the genes *GCK, HNF1A, HNF4A*, or *HNF1B* during this period, allowing a comprehensive assessment of the state of monogenic diabetes testing within New Zealand. Test requests were performed either as a single gene test or small multi-gene panel test (any combination of the 4 genes listed above, performed in tandem).

In addition, requests sent to the Exeter laboratory were for either the neonatal diabetes gene panel or monogenic diabetes gene panel, both large panels as detailed below. Data on these referrals to the Exeter laboratory was available for patients sent from all regions across NZ. Requests for Monogenic Diabetes gene testing sent to laboratories other than Exeter were not captured but would be expected to be rare. Mitochondrial diabetes (MIDD) testing was not captured, as this was offered by another laboratory (Christchurch).

Referrals included a testing algorithm checklist (4, 5) where referrers indicated if single gene, small multigene, or large panel testing was requested, or cascade testing of a known gene variant. Whether patients had single gene testing, small multigene, or large panel testing, or any follow up testing was dependant on the clinician and hence this varied between patients with no standard pathway, other than the guidance detailed above.

Testing method at LabPlus was either full gene Sanger sequencing; cascade testing of a known familial variant (i.e. single exon Sanger sequencing only); or copy number (dosage) analysis using comparative genomic hybridisation (CGH) array.

For Sanger sequencing, the coding regions of the gene and flanking +/- 20bp (encompassing the splice sites) were sequenced. Analysis of sequence data was performed using Variant Reporter Software. Minimum sequence trace Phred score was 35, corresponding to an average false base call frequency of 0.031%.

Copy number analysis was performed using an Agilent 60K Custom CGH array for exonic deletions and duplications. Agilent CytoGenomics software was used for analysis. The inclusion threshold for analysis of a dosage change required a log2ratio of less than -0.25 over at least 3 contiguous probes for a deletion, and a log2ratio of greater than 0.25 over at least 3 contiguous probes for a duplication.

The Exeter Genomics Laboratory performed genetic testing for 27 monogenic diabetes genes that included the *MT-TL1 m.3243A>G* and 26 other autosomal diabetes genes (*ABCC8, CEL, CISD2, GATA4, GATA6, GCK, HNF1A, HNF1B, HNF4A, INS, INSR, KCNJ11, LMNA, NEUROD1, PAX6, PCBD1, PDX1, PLIN1, POLD1, PPARG, RFX6, SLC29A3, TRMT10A, WFS1, ZBTB20, ZFP57).* Patients under 12 months were tested for 30 neonatal diabetes genes *(ABCC8, BSCL2, CISD2, CNOT1, COQ2, EIF2AK3, FOXP3, GATA4, GATA6, GCK, GLIS3, HNF1B, IER3IP1, IL2RA, INS, INSR, KCNJ11, LRBA, MNX1, NEUROD1, NEUROG3, NKX2-2, PDX1, PTF1A (coding and distal enhancer region), RFX6, SLC19A2, SLC2A2, STAT3, WFS1, ZFP57).* The coding regions, 50 nucleotides of flanking intronic sequence of these genes and the mtDNA nucleotide m.3243 were analysed for single nucleotide variants (SNV), indels and gene deletions by targeted Next Generation Sequencing (tNGS). This assay did not target any other mitochondrial mutations or structural rearrangements. The Agilent SureSelect custom capture library and an Illumina NetSeq 500 NGS sequencing platform was used, according to the methodology described by Ellard et al. (6). The assay sequenced 99.7% of bases within the regions of interest at a minimum 30x read depth for all patients. Copy number variant (CNV) analysis was performed using ExomeDepth according to the methodology described by Parrish et al. (11). The estimated sensitivity for CNV detection was >95%. Neonatal diabetes cases also had Methylation-Specific Multiplex Ligation-Dependent Probe Amplication (MS-MLPA) for the 6q24 differentially methylated region (*PLAGL1* DMR) using MRC Holland kit ME033.

Interpretation and classification of variants was based on the American College of Medical Genetics and Genomics (ACMG) guidelines (from July 2015 onwards) and on best available evidence prior to this. Results were classified by ACMG guidelines and reported as no variant detected (class 1-2 only), variant of uncertain significance (VUS, class 3, C3) or variant detected (likely pathogenic or pathogenic, class 4 or 5, C4/5 variant). Variants were described using the Human Genome Variation Society (HGVS) nomenclature guidelines. Results were classified as “positive” if a class 4 or 5 variant was identified, and “negative” or “normal” if a class 1, 2, or 3 variant were found.

Ethnicity data for all patients was coded using the Stats NZ classifications system, as European (Group 1), Māori (Group 2), Pacific Peoples (Group 3), Asian (Group 4), Middle Eastern/Latin American/African (MELAA) (Group 5) or other (Group 6 and 9). Respondents identifying as ‘Fijian Indian’ were coded as Asian; responders identifying as “New Zealander” were aggregated as European, as per the NZ ethnicity Data Protocols for the Health and Disability Sector. Prioritised output was used to assign those with multiple ethnic affiliations, with Māori and Pacific codes prioritised over European.

### Ethics

The study was assessed as being low risk and out of scope of HDEC (Health and Disability Ethics committees). Approval for the audit was granted by the ADHB research board (reference 7581).

## Results

In total, 453 individual patients were tested at Auckland, of which 371 had proband/full gene sequencing (as opposed to cascade or dosage testing). A further 116 tests were performed at Exeter, of which 10 were the neonatal diabetes panel. Data was not available to determine which of the patients tested at Auckland went on to have further extended panel testing at Exeter, nor how many patients were sent straight to Exeter.

### Single gene request vs panel testing

**Figure 1:**
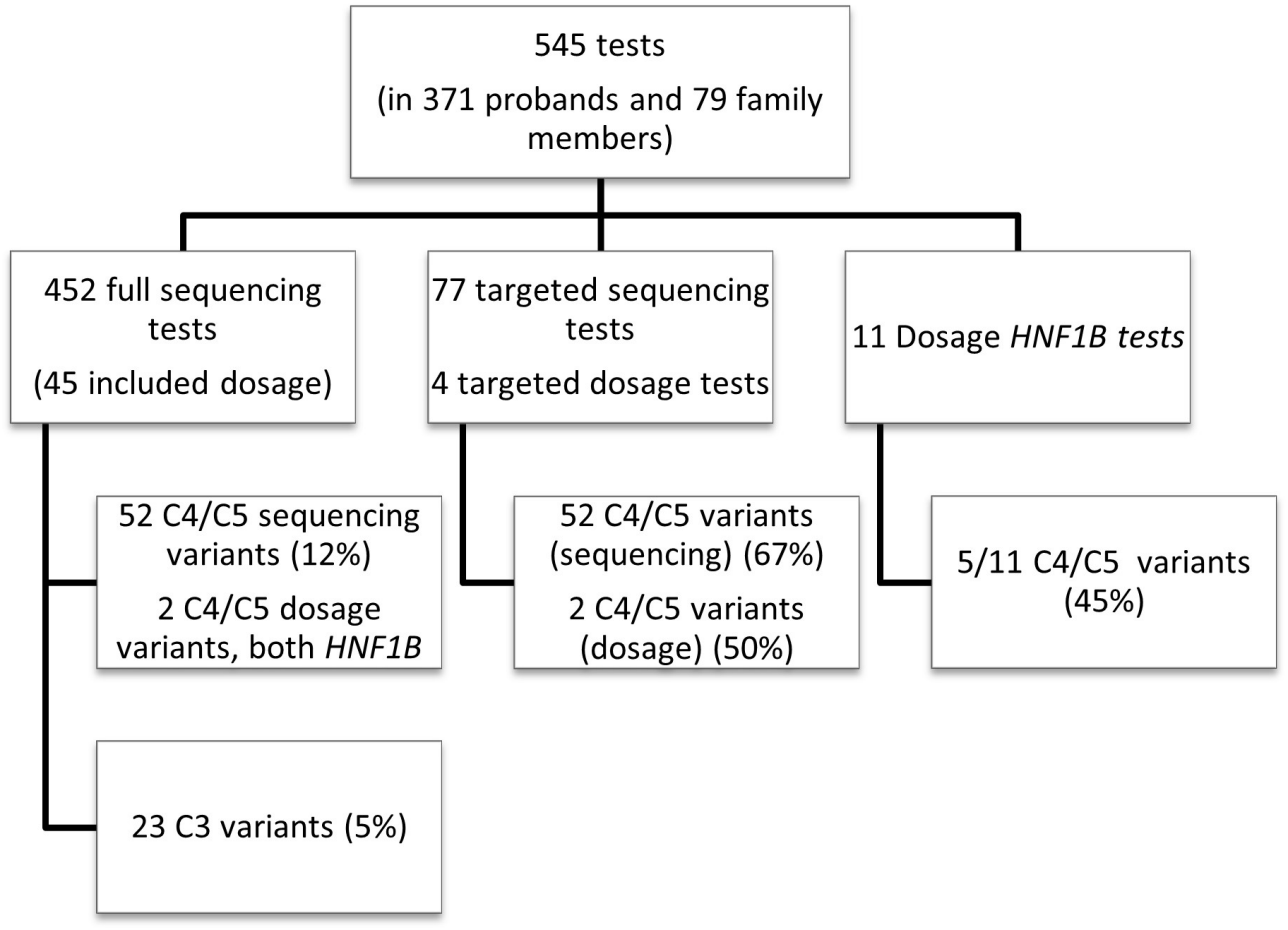
Overall test breakdown. Overview of the LabPlus testing for monogenic diabetes January 2014 – December 2020, using Sanger sequencing, and CGH array for dosage testing. 116 tests were also sent to Exeter for wider panel testing using NGS. C4/C5 = likely pathogenic/pathogenic variant, as defined by ACMG. C3 = Variant of uncertain significance, as defined by ACMG.

545 tests in total were performed during the seven year period at LabPlus (**Figure 1**): most (83%) were sequencing tests, either as single (75%) or small multi-gene tests (25%); only 11 (2%) were dosage tests for *HNF1B*, and 81 (15%) were cascade tests for familial follow up. Dosage analysis was also performed for 45 probands in addition to sequencing (45/452), and 4 of the cascade tests were for dosage.

**Figure 2:**
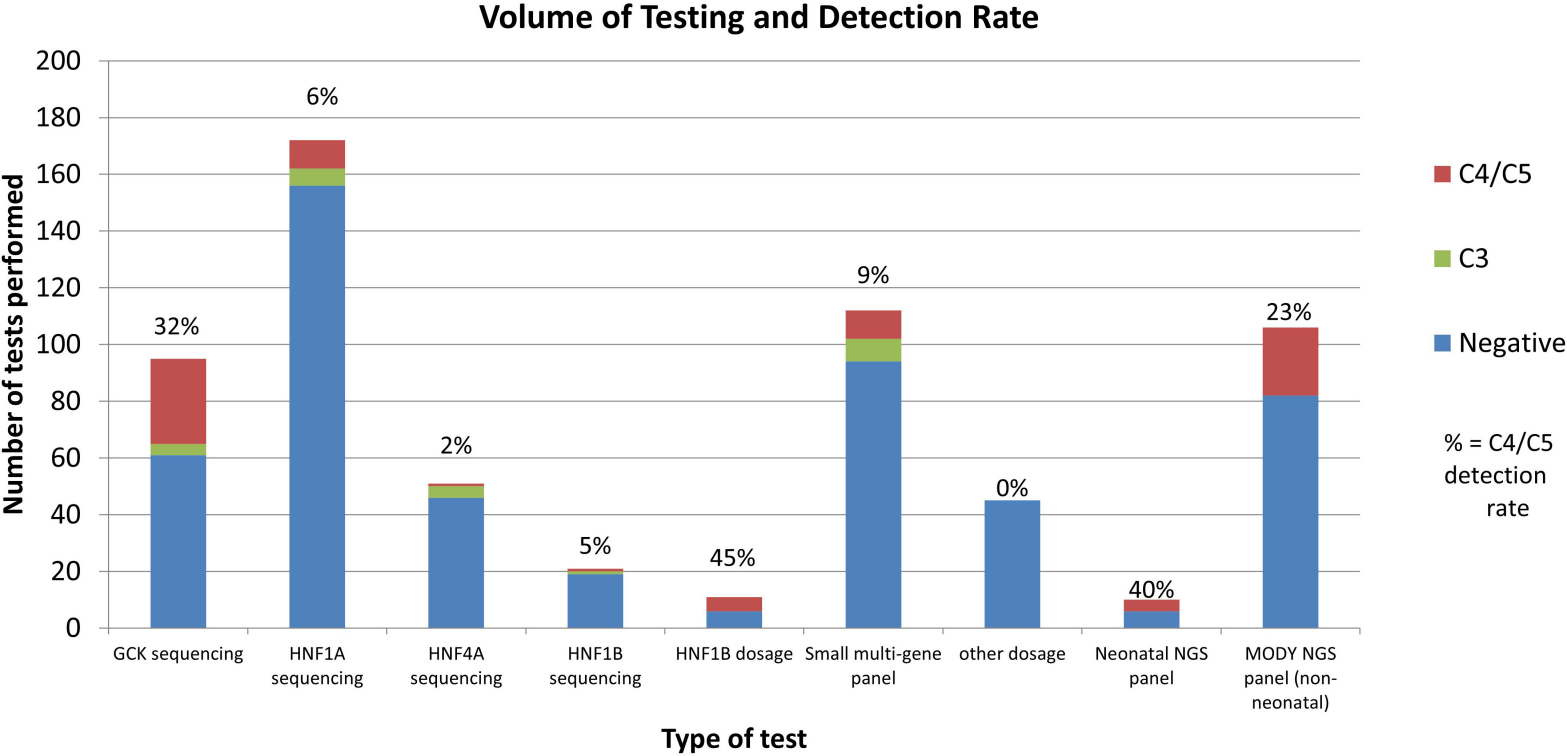
Volume and Detection Rate by test type. Other dosage: dosage analysis performed for genes other than *HNF1B.* MODY NGS panel: Exeter large NGS panel testing. Negative indicates no C3/C4/C5 variants found.

Using single gene testing or small multi-gene panel testing, 52 (12%) of sequencing tests in probands received a diagnostic result (C4/5 variant), and 23 (5%) received a VUS. The detection rate varied significantly by gene (**Figure 2**). Dosage testing of *HNF1B* gave the highest diagnostic detection rate, with 45% of tests with a C4/5 variant (5/11 tests). The next highest detection rate for sequencing was *GCK*, with class 4/5 variants found in 32% (30/95) of tests. Of 95 requests for single gene testing for *GCK*, 43 came from females of child-bearing age (defined as 16-45yrs), of which 16 had a C4/C5 variant (37% detection rate). Twenty-five requests came from the paediatric population, of which 7 (28%) had a C4/C5 variant. Twenty-seven tests therefore were not from reproductive-age female or paediatric patients, of which 7 were C4/C5 (25%), indicating a high detection rate for this gene in all cohorts tested.

*HNF1A* had a C4/5 detection rate of 6% (10/172 tests); *HNF1B* sequencing detected only one C4/5 variant in 21 tests (5%). The lowest detection rate was *HNF4A*, with 1 C4/C5 in 51 tests (2% detection rate). However, *HNF4A* gave the highest rate of VUS (8%). Seventy-three patients had follow up testing after their first single gene test was normal. Of the follow up tests performed, most (62/81, 77%) were for further single gene requests, and 19 (23%) were for multi-gene testing. Only 3 follow up tests were positive (4%), although 5 tests gave a VUS.

Requests for small multi-gene panel testing (any combination of the 4 available genes) gave a detection rate of 9% C4/C5 (10/112).

Comparatively, of the 106 who had general monogenic diabetes gene panels performed at Exeter using the 27 gene NGS panel, 24 (23%) received a C4/C5 result. 18 of these cases were in *GCK, HNF1A, HNF4A*, or *HNF1B* (16% detection rate). 6 cases were found in genes not available at LabPlus (*ABCC8* (2 cases), *KCNJ11*, *WFS1* (2 cases) and m.3243A>G). Of the 10 neonatal gene panels performed, 4 were positive (40% detection rate), with C4/C5 findings in *INS, FOXP3, KCNJ11* and methylation 6q24 abnormalities.

Dosage analysis was also performed for 45 of the gene tests at LabPlus, performed at the laboratory’s discretion, for genes other than *HNF1B*. No other copy number variants (CNVs) were detected. One known copy number variant in *HNF1A* was analysed as cascade testing.

Cascade testing had a high detection rate (67%, 52/77 sequencing tests giving C3/4/5 variants).

### MODY probability scores in those who were referred for genetic testing

**Figure 3:**
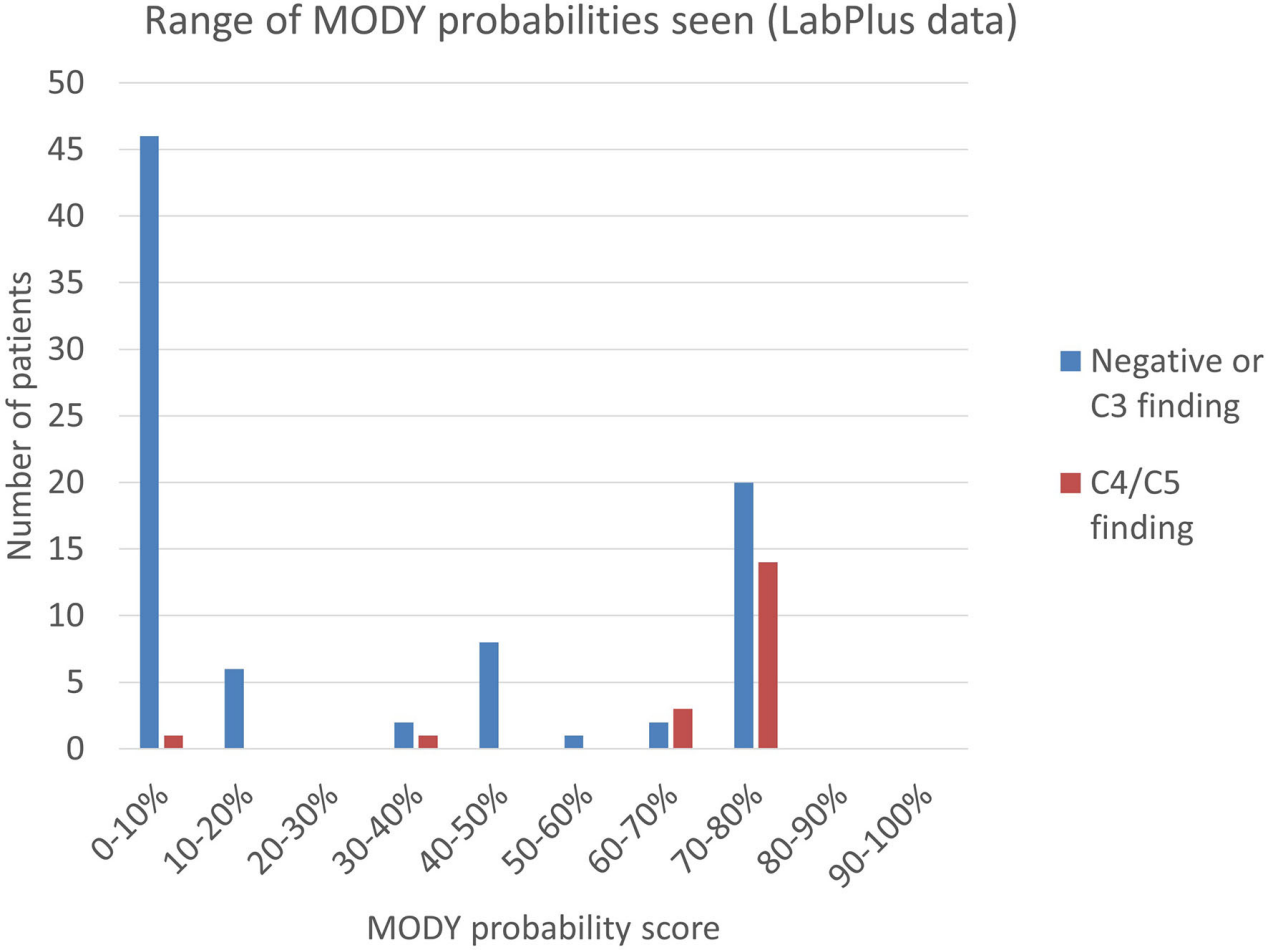
Range of MODY probability scores seen using the MODY pre-test probability calculator. MODY pre-test probability of patients referred to LabPlus, where sufficient clinical data was available (n=104); calculations as per Shields et al. (7).

Clinical data to calculate a MODY pre-test probability score was available on 115 of the 371 patients from the LabPlus data, but only 104 were diagnosed with diabetes under 35 years and suitable for use with the MODY calculator. 1 result was not available. The range of MODY probability scores in the 104 patients was from 0.7% - 75.5% (**Figure 3**). There were 19 C4/C5 results in this cohort (18%), with MODY scores of 8.2% -75.5%. One patient scored 8.2%, one 35.8%, and all others with C4/C5 variants scored >60%. 53 patients (51%) scored <20%. Two of these patients had a VUS, scoring 4.6% and 7%. Only one patient with a C4/C5 variant scored <20%, and this patient had a sequencing variant in *HNF1B*, and single gene sequencing was performed based on the phenotype.

The MODY probability score of patients with C4/C5 variants in the Exeter laboratory data was examined. No data was available on detection of C3 variants in the Exeter cohort. 43 patients had sufficient data to calculate the MODY pre-test probability score and were in patients diagnosed between 1-35 years. 14 of these gave C4/C5 results (33%) and the MODY scores of positive patients were between 4.0% - 75.5%. 14 patients (33%) scored <20%, of which two had C4/C5 variants: one patient scored 4.0% and had a *HNF1A* variant; another patient scored 12.6% and had a *HNF4A* variant. All remaining C4/C5 patients scored above 49.4%.

Combining the LabPlus and Exeter data, out of 147 patients with a MODY probability score calculable, 67 (45%) scored <20%.

Average MODY probability in Europeans was 34%, n=48; in Māori, it was 41%, n = 21; Pacific = 27%, n =18, and in Asians was 46% (n=16), (**Table 1**, LabPlus data only). Only one patient was classified as MELAA. The number of patients scoring <20% was slightly higher in the Pacific population (61%) of patients.

**Table 1:** MODY pre-test probability by ethnicity.

	European	Māori	Pacific	Asian
Patients with MODY pre-test probability data and ethnicity (n=104^*^)	48 (46%)	21 (20%)	18 (17%)	16 (15%)
Average MODY probability of all patients	34%	41%	27%	46%
Average age of patients tested	30	23	15	23
Number of patients with C4/C5 results	11/48 (23%)	5/21 (24%)	1/18 (6%)	2/16 (13%)
MODY probability range for positive tests	8.2%-75.5%	62.4-75.5%	75.5%	75.5%
Number of patients scoring <20%	25/48 (52%)	9/21 (43%)	11/18 (61%)	7/16 (44%)
Number of patients scoring <20% with a C4/C5 variant	1	0	0	0

^*^Note one patient of ethnic classification MELAA (Middle Eastern/Latin American/African).

### Testing frequency by ethnicity

**Figure 4:**
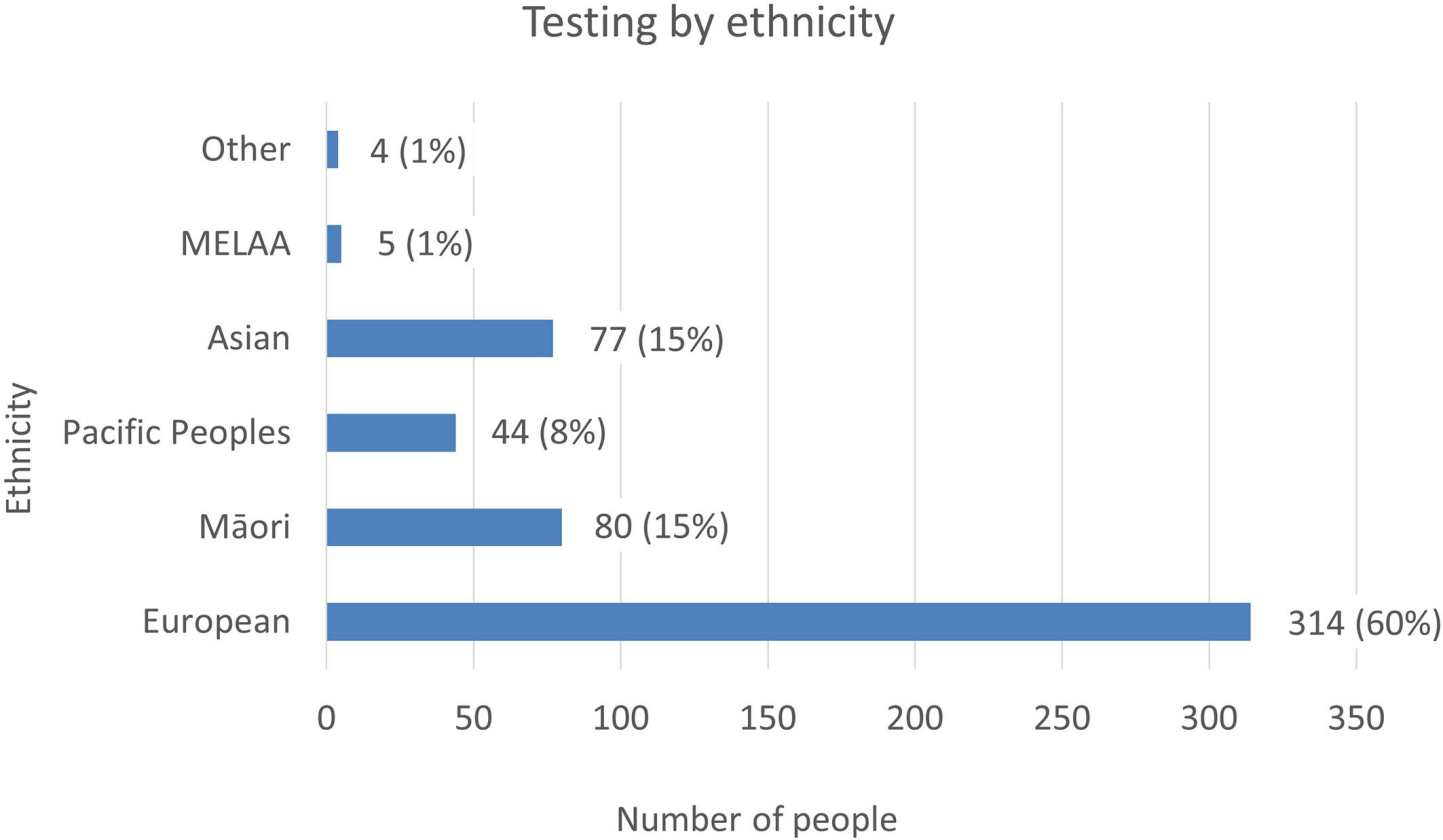
The number of tests referred to LabPlus and Exeter, by ethnicity. % Indicates the proportion of testing each ethnicity represents of the total cohort.

Ethnicity data was available on all patients referred to LabPlus and 71 of the patients referred to Exeter. The proportion of referrals by ethnicity was reviewed (**Figure 4**). European referrals made up the majority, at 60%, Māori and Asian referrals were 15% each, and Pacific Peoples were 8%.

National data on ethnicity and diabetes lists a total of 277,803 individuals with a diabetes diagnosis in NZ in 2020, with Pacific people making up 14% of cases (38,433 individuals), Indian 8% (21,146 individuals), Māori 16% (45,637 individuals), and European 62% (172,587 individuals) (12). People of Pacific ethnicity are known to have a higher prevalence of type 2 diabetes, with a prevalence of 114.9/1000, compared to 100/1000 in Indian, 67.7/1000 in Māori and 29.1/1000 in Europeans (12). Given the higher prevalence of diabetes in Pacific people, but the lower proportion of monogenic tests this population represents, the prevalence of monogenic testing was lowest in Pacific people. However, this may be appropriate, given the higher rates of obesity and type 2 diabetes in this population. Testing rates by ethnicity were 0.1% Pacific (44 tests/38,433 diabetes diagnoses), 0.4% Asian (77/21,146), 0.2% Māori (80/45,637), and 0.2% European (314/172,587), indicating generally low testing rates in all ethnicities.

Of the 371 patients undergoing full sequencing at LabPlus, detection rates of C4/C5 variants were 40/206 (19%) for European, 9/61 Maori (15%), 1/34 Pacific (3%), 3/59 Asian (5%).

## Discussion

The detection rate for single gene testing and small multi-gene panels was relatively low, particularly compared with the detection rate of the NGS non-neonatal diabetes large gene panel used by Exeter (12% vs 23%). *GCK* sequencing, *HNF1B* dosage, and cascade testing offered the highest detection rates. Follow up testing after a negative single gene test was also mostly uninformative, with a detection rate of 4%, indicating the sequential single gene approach is inefficient. The referral rate for testing in patients identifying as Māori was similar to population data. However, Pacific people with diabetes were less likely to be selected for monogenic diabetes testing, compared to other ethnicities. Pacific people also had lower detection rates of monogenic diabetes (3% C4/C5 rate). The prevalence of diabetes by age shows the higher prevalence of obesity related type 2 diabetes in Pacific people, and hence the lower proportion of monogenic diabetes in this ethnic group are likely to be factors underpinning both reduced referral and diagnostic rates (13). However, it may represent other complex factors affecting patients’ presentations, interaction with healthcare providers, or clinician bias.

The prevalence of monogenic diabetes has not previously been reported in NZ. Compared to the Virtual Diabetes Register (VDR) data, (which lists 277,803 people with diabetes in NZ in 2020), the roughly 569 patients tested over this 7-year period represent just 0.2% of people with diabetes. If 1% prevalence is expected (a conservative estimate), this indicates a need to significantly increase testing rates. In other countries, work shows that the more testing is available, the higher the estimated prevalence of monogenic diabetes becomes (8).

Our findings are consistent with other countries’ data that *GCK* and *HNF1A* are the most common forms of monogenic diabetes. However, in the UK, *HNF1A* is more common (52%) than *GCK* (32%) (2) whilst in the many other countries, *GCK* is the most common (14–17). The reason for this difference is unknown – our finding that *GCK* is the most common form in NZ may either be a true difference to the UK population, or may indicate that the NZ testing strategy is resulting in easier access to *GCK* testing e.g. for pregnant women with gestational diabetes and children with hyperglycemia who are more often under specialist care, with insufficient testing of other patients with diabetes who are more often under primary care.

The high detection rate of *HNF1B* dosage testing (45%) likely reflects the more specific phenotype of *HNF1B* deletion patients, with syndromic and extra-diabetic features, and indicates single gene testing may still have a role in this setting. The MODY calculator is not validated for detection of *HNF1B* variants (7). Instead, the HNF1B score is recommended as a score of <8 can rule out *HNF1B* with 98% sensitivity, and a negative predictive value of >99% (18).

Dosage analysis of genes can generate significant extra workload for a laboratory, particularly in the single gene testing setting. Copy number variants (CNVs) have been documented to occur in the common MODY genes other than *HNF1B* but at low prevalence [1.2% *HNF1A*, 1.9% *HNF4A*, and 1.8% of *GCK* (19–21)]. In our study, in the small cohort of probands who received dosage analysis for genes other than *HNF1B*, no CNVs were found. This indicates that whilst dosage analysis is desirable, the added benefit to including this as routine for all patients is not high.

### Cascade testing

The cascade tests had a very high detection rate (67%) - as expected given the pre-test probability for these tests will be 50% or higher. However, only a small number of cascade tests were performed (77 cascades, compared to 452 probands), around 1 in 6 probands generating a cascade test. Each positive proband should be expected to generate several cascade tests for other family members, who may benefit from better treatment, better complication management and improved pregnancy management. In the USA, 307 probands with monogenic diabetes generated 362 cascade tests (17), indicating NZ cascade testing for monogenic diabetes is significantly lacking. Cascade testing is clearly cost-effective with a high detection rate and is cheaper than full/multiple gene Sanger sequencing, as only one Sanger amplicon is required, with reduced interpretation time, hence should be used wherever there is a known familial variant. Reasons for this low rate of cascade testing may include a lack of resources to systematically track family members and offer genetic testing and counselling; perceived lack of benefit to testing, from either clinicians or family members; or difficulties for relatives and clinicians in accessing suitable referral pathways.

### MODY calculator

The MODY probability data shows that using the MODY probability calculator to exclude patients from testing if scoring <20% would have reduced testing by 45% (67 test requests). However, three patients who scored <20% had C4/C5 variants. This is expected for the MODY probability calculator, as finding 3/67 patients with C4/C5 variants is consistent with the <20% pre-test probability of having MODY in these individuals. One patient had an *HNF1B* phenotype, for which the MODY calculator is not validated. The other two patients were insulin treated, and the prior probability for MODY is much lower in this group, since type 1 diabetes is the most common diagnosis in such patients. Hence pre-test scores of >10% are currently recommended in the UK to select patients for testing who are on insulin, after triage tests of retained C-peptide and negative antibodies. In validating their MODY probability calculator, Shields et al. suggested a pre-test MODY probability of >25% be used in patients not treated with insulin within 6 months of diagnosis, and >10% in those treated with insulin within 6 months, due to the impact of finding a pathogenic variant being more significant in those on insulin.

A limitation is that most patients tested only received a single gene test, and hence we cannot exclude a monogenic cause that may have been uncovered if a broader gene panel had been used. Furthermore, data for MODY pre-test probability was only available on a subset of patients referred for testing (104/371, 28%, of LabPlus patients, and 43/106 (41%) of Exeter patients).

The MODY probability score is not suitable for those requiring cascade testing or those with a syndromic phenotype such as those consistent with *HNF1B*, MIDD, or a severe insulin resistance phenotype.

Those with Māori ethnicities had similar MODY probability scores compared to Europeans, however those of Pacific ethnicity had lower MODY probability, reflecting the lower diagnostic yield in this ethnic group. Further studies, particularly of the use of biomarkers to help enrich the testing population, and on the prevalence of monogenic diabetes in the Pacific population are required. The number of patients with insufficient clinical data, and the significant amount of clinical data required to calculate the MODY pre-test probability could mask inequalities in the use of this calculator in certain ethnicities.

### Design of a NZ testing pathway

**Figure 5:**
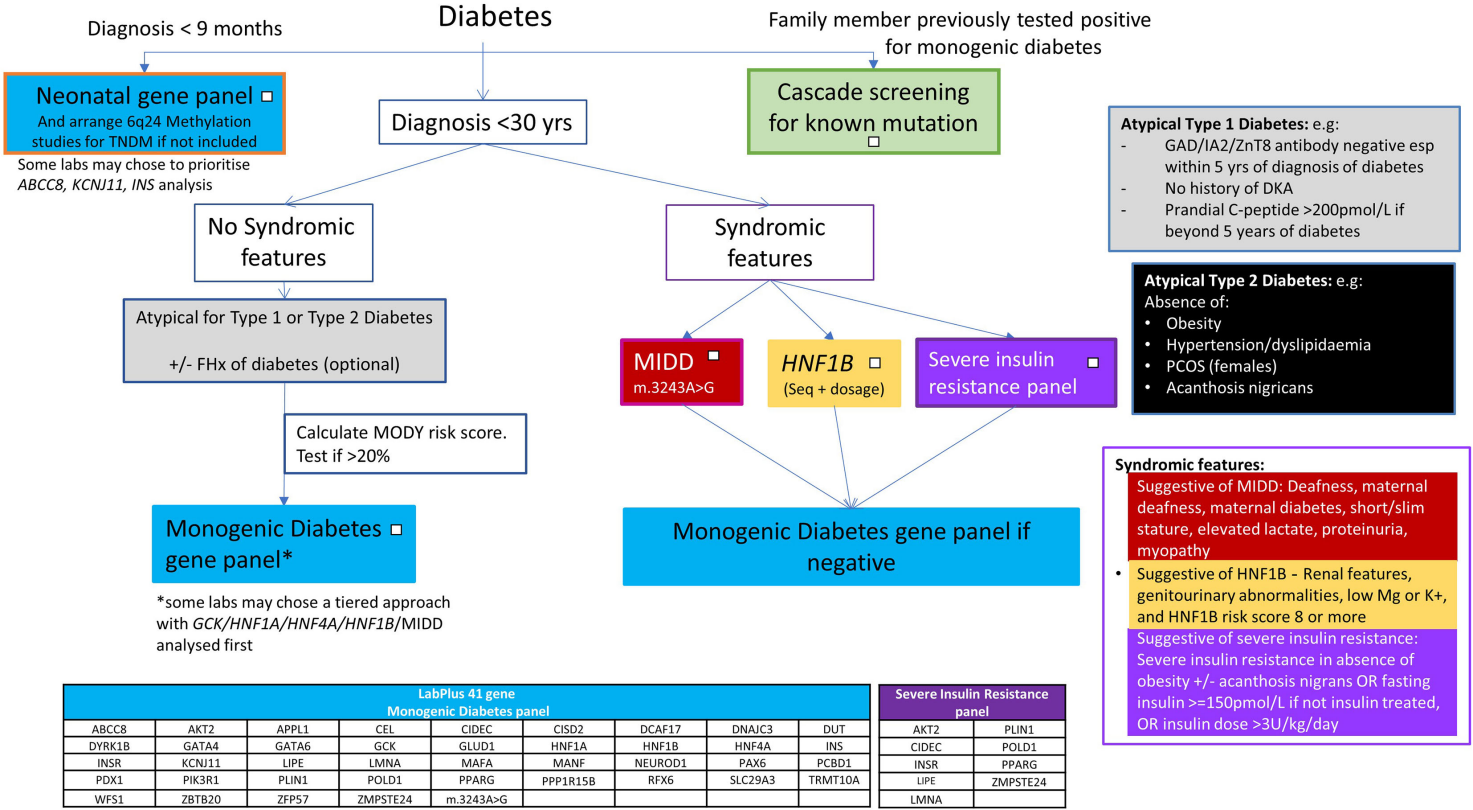
New monogenic diabetes testing algorithm for NZ.

Most centres aim to keep a detection rate of above 20%, to be cost effective, although an overall detection rate of over 30% in monogenic diabetes testing has been suggested to be cost saving (22, 23). The single gene testing pathway for NZ (5) was designed to keep Sanger sequencing costs to a minimum. The low detection rate (12%) of the current testing approach, compared to the improved detection rate (23%) of the large NGS panel approach indicates sequencing all monogenic diabetes genes simultaneously through the use of an NGS panel may give a higher detection rate. A 41 gene NGS based panel has been introduced at the Auckland laboratory and the data presented here have been used to guide how to use this panel, using the flowchart (**Figure 5**) as part of new national guidance from the New Zealand Society for the Study of Diabetes (NZSSD). In patients diagnosed within the neonatal period, the neonatal NGS panel remains the recommended genetic test. In patients diagnosed with diabetes beyond the neonatal period, requestors are asked to look for features of atypical Type 1 or Type 2 diabetes mellitus and consider a limited number of clear syndromic presentations (HNF1B, MIDD, severe insulin resistance), in which case single gene testing may be justified. Otherwise, requestors are prompted to calculate the MODY pre-test probability, and proceed to broad gene panel testing if the pre-test probability is >20%. The use of a broad gene panel in this situation replaces the sequential single gene approach recommended previously.

### Gene panel content

The genetic content of the monogenic diabetes NGS panel used is recommended to include at least the 5 common diagnoses (*GCK, HNF1A, HNF1B, HNF4A*, and m.3243A>G). Given the detection rate for the Exeter panel increased from 16% to 23% when including other syndromic genes, there appears to be benefit in offering a more extensive gene panel. Syndromic forms of diabetes are estimated to be found in 18% of monogenic diabetes cases, including patients presenting without syndromic features (as features are either undiagnosed or undocumented). Additionally, including syndromic genes in the routine gene panel allows clinicians to focus on identifying patients for testing, rather than which type of monogenic diabetes, given the overlapping clinical presentations of each (24). Whilst *HNF1B* and MIDD make up the majority of syndromic cases, *WFS1* is the third most common syndromic form reported (15).

The need to detect syndromic or rarer causes of monogenic diabetes must be balanced against the increased costs and analysis time of analysing rarer genes, and the diminishing returns that are found when increasing the size of the gene panel offered. Therefore, whilst offering an extended panel gives the highest detection rate, the specific protocol a laboratory takes for analysis will depend on the set up of the local infrastructure and testing cohort. Some laboraties may choose to use a tiered approach, with initial analysis of the common genes first, with extension to the remaining genes if negative, or initiate full panel testing in all patients, depending on the logistics and set up of their sequencing pathway.

Ultimately the final question of which test is most cost effective depends on the price of testing – a cost which will vary by laboratory. Sanger sequencing, with the need to amplify each exon, is slow and becomes expensive when multiple genes are sequenced, particularly compared to the cost efficiencies achieved when performing an NGS panel. This means that the cost of Sanger sequencing the common monogenic diabetes genes is often more expensive than using a wider NGS panel. There are also other clinical benefits to using a wider panel of testing from the outset - clinicians and laboratories do not have to re-activate further testing, benefitting continuity of care, clinician and patient time and reducing the risk of losing patients to follow up.

### Neonatal presentations

The left side of this flowchart, for those with neonatal onset diabetes, remains the same as the previous algorithm - prompting a NGS gene panel of all genes associated with neonatal diabetes (25, 26). *ABCC8, KCNJ11* and *INS* are the most commonly involved genes in this age group and maybe present on a generic monogenic diabetes panel. However, other neonatal forms e.g. *FOXP3, STAT3, LRBA, EIF2AK3*, are likely to require a specific neonatal panel to include coverage (27). Methylation testing for 6q24 imprinting disorders should also be performed in this age group as anomalies at this locus account for 60-70% of transient neonatal diabetes (27, 28).

In paediatric presentations above 9 months old, extensive gene panel testing is to be encouraged, to identify monogenic diabetes early, particularly the syndromic forms that direct clinical management, and clinicians should follow the central part of the pathway for atypical type 1 and type 2 diabetes presentations.

### Syndromic forms

The more specific phenotypes of MIDD, *HNF1B* and insulin resistance mean single gene/specific insulin resistance panel testing may still be appropriate for these clinical presentations. The high detection rate for *HNF1B* supports the single gene approach to be suitable for this phenotype. No data is available on MIDD and insulin resistance panel testing, and further work will need to review the appropriateness of these testing pathways. The MIDD phenotype can be caused by a range of different mutations in mitochondrial tRNA, but the m.3243A>G variant is estimated to account for 85% of cases (29). If there is a strong clinical suspicion of a mitochondrial form, specific testing for other mitochondrial variants should be arranged. Severe monogenic forms of insulin resistance are under-recognised and typically present without obesity, unlike the polygenic insulin resistance associated with obesity (30).

### Education

Increasing awareness of monogenic diabetes is known to increase the detection rate, hence improved education and access to diabetic nurse specialists are to be encouraged. This is particularly so as test requests are only accessible through secondary care which, especially for patients with non-type 1 diabetes, is limited. Better access to cascade testing is also required to increase uptake and referral rates (8, 31).

Wider testing will generate more variants of uncertain significance, variants in low penetrance genes (32) and dual presentations of type 2 diabetes mellitus, and clinicians must be aware of how to interpret such results.

## Summary

Data from 7 years of monogenic diabetes testing in NZ suggests more testing is to be encouraged, as testing rates are low, particularly for cascade genetic testing. A new testing strategy is proposed using an NGS based panel approach to improve diagnostic detection rates. A MODY probability score of >20% is proposed to be used to prioritise cost-effective selection of patients who are most likely to benefit from testing. Where a C4/C5 variant is identified, the patient and family should be referred for genetic counselling, to enable cascade testing of family members, an aspect which is currently significantly under-utilised.

## Data availability statement

The raw data supporting the conclusions of this article will be made available by the authors, without undue reservation.

## Ethics statement

The studies involving human participants were reviewed and approved by the ADHB (Auckland District Health Board) research board (reference 7581). The study was assessed as being low risk and out of scope of HDEC (Health and Disability Ethics committees). Written informed consent from the participants’ legal guardian/next of kin was not required to participate in this study in accordance with the national legislation and the institutional requirements.

## Author contributions

RM and FH designed the study. FH analysed the data and prepared the manuscript. MG reviewed the manuscript and provided laboratory input. KC supplied data from Exeter. RP and RM reviewed the manuscript and provided clinical input. RM provided overall supervision. All authors contributed to the article and approved the submitted version.
